# Supplementary material for: Māori Experiences and Beliefs about Antibiotics and Antimicrobial Resistance for Acute Upper Respiratory Tract Symptoms: A Qualitative Study
Source: Antibiotics (Basel). 2022 May 26;11(6):714. doi: 10.3390/antibiotics11060714 (PMC9220347; doi:10.3390/antibiotics11060714)
Supplement: Supplementary file 1 [file antibiotics-11-00714-s001.zip › antibiotics-1624930-supplementary.pdf]

Supplementary Materials

File S1: Definitions of Te Reo terms

Aotearoa = New Zealand

Iwi = tribe, people

Māori = indigenous people of New Zealand

Marae = open space in front of the wharenui (meeting house); a communal or sacred place where values and philosophy are reaffirmed

Moko = grandchild

Kōrero = tell, say, speak, read, talk, address

Pākeha = New Zealanders primarily of European descent

Papakāinga = home

Reo = language

Rongoā – Māori medicine

Tikanga = correct procedure, custom

Te Ao Māori = the Māori world

Te Tiriti o Waitangi = Māori document of the Treaty of Waitangi

Tohunga = esteemed holders of expertise including healing

Wairua = spirit, soul

Whakaaro = thoughts, ideas

Whānau = family

File S2: Discussion Guide

1. **Introduction**

   Introduce self, offer opportunity to open with karakia, study purpose, rights (go over Consent Form).

Tell me about you (pepeha)

- What language is spoken at home?
- What are your hobbies / work?

2. **Relationship with GP**

   Tell me about your relationship with you GP?

   - How would you describe your relationship with your GP? Positive or negative?
   - Are they easy to access?
   - How much influence do you feel you have over the decisions that your GP makes about your health?

3. **General discussion about GP consultation**

   Thinking back to a time when you last saw the GP and you were prescribed antibiotics…

   Probes –

   - How did you feel?
   - How come?
   - Did the GP offer antibiotics?
   - Did you ask for antibiotics ? Why /why not?

   Were there times when you were at the GP's office when you asked for antibiotics but was **not** prescribed by your GP…

   Probes –

   - What happened?
   - What did the GP say or do?
   - How did you feel? How come?
   - What did you do?
   - Did you visit another GP to get antibiotics? Why / why not?

Has your GP given you a prescription of antibiotics and said don't get it dispensed now, only get the prescription filled if you get worse ?

Probes –

- What happened?
- How did you feel?
- Did you get your prescription dispensed? Why/why not?

4. **Views about antibiotics**

   Tell me about antibiotics – thinking back to the last time you had antibiotics:

What comes to mind?

Probes –

- Feelings, images, colours, pictures, associations
- Any associations with cold/flu – why?
- Viruses / bacteria / germs / ‘bugs’ – why?

Tell me about the last two times you were prescribed antibiotics

- What happened? Did you feel you needed antibiotics?
- What did you say?
- What did your GP say?

Do you have any trouble accessing antibiotics when you want?

Probes –

- Can you tell me what happened?

We know it costs money and time to book an appointment with your GP and to get antibiotics when you are sick. Thinking back to the last time you had cold/flu symptoms, what influenced your decision whether or not to see your GP?

Probes –

- What symptoms did you have?
- How did you feel?

When do you expect to be prescribed antibiotics?

Probes –

- Do you expect to have antibiotics when you really sick? What symptoms?
- Do you expect antibiotics when you have been sick for a long time? How long?
- How do you feel before you start feeling like you need antibiotics?
- Do you think antibiotics will help?

Were there times when you asked for antibiotics and the GP resisted at first?

Probes –

- What happened?
- Why did you ask to receive antibiotics ?
- What did you say?
- What did the GP say?
- How did you feel?

Do you have any whakaaro on antibiotics ?

Probes –

- What are the good things / benefits?
- What are the bad things / harms?

- Do you think antibiotics are useful for all situations? Which ones are they useful / not useful for?

Where have you heard information about antibiotics?

Probes –

- People / social media / leaflets / news / online
- What did you hear?
- What was the key message?
- How did it make you feel?
- Was it relevant to you? Why / why not?
- How did it make you feel about antibiotics – did it persuade you that antibiotics were good / bad? How come?

5. Providing information to support safe and effective use of antibiotics

We want to make sure that we provide information in a way that ensures antibiotics are used safely and effectively. One thing we want to make sure is that antibiotics are only used when they are necessary – for example, antibiotics are not useful for cold/flu because they are caused by viruses not bacteria.

What do you think of this?

Probes –

- What do you think are the differences between viruses/ bacteria?
- What happens if antibiotics are given for infections caused by viruses

What are the things that you think need to happen for people not to request for antibiotics from their GP?

Probes –

- What information would help?
- Who should give the information?
- In what way?
- What might their reaction be to the information?
- What may help people be more open to these messages?
- What may cause people to disagree or ignore the messages?
- What groups of people do you think may respond better? How comes?

6. **Culture**

Do you think your culture relates to use of antibiotics?

- Influence of rongoā and antibiotic use?

How can Māori culture and beliefs aid antibiotic use for Māori?

- Would it be useful to have a cultural interface to help you understand your medicines ?
- Kaiatawhai / Māori cultural support
- Interpreters
- Information in Te Reo

7. **Conclusion**

Is there anything you'd like to share about your experiences with receiving/obtaining antibiotics that you think would help us to provide a better service to other patients?

Table S1. Demographics of participants

	Long term condition	Regular medication	Highest qualification	Help with daily activities	Managing medication	Living situation
P1	Yes – Gout	Yes	Trade/Certificate	None	Self – manage	Extended whānau
P2	No	No	University	None	Self-manage + healthcare professionals	Immediate whānau
P3	Did not complete	No	Trade/Certificate	Some assistance	Self-manage	Immediate whānau
P4	No	No	High School	Some assistance	Self-manage	Immediate whānau
P5	Did not complete	Did not complete	Did not complete	Did not complete	Did not complete	Did not complete
P6	Did not complete	Did not complete	Did not complete	Did not complete	Did not complete	Did not complete
P7	Yes – Asthma	Did not complete	High School	Did not complete	Self-manage	Immediate whānau
P8	Did not complete	Did not complete	Did not complete	Did not complete	Did not complete	Did not complete
P9	Yes-Rheumatoid Arthritis	Yes	University	Did not complete	Self-manage	Extended whānau
P10	Did not complete	Did not complete	Did not complete	Did not complete	Did not complete	Did not complete
P11	No	Yes	Did not complete	Some assistance	Self-manage	Immediate Whānau
P12	Yes - Osteoarthritis	Yes	High School	None	Self-manage	Immediate whānau
P13	Did not complete	Did not complete	Did not complete	Did not complete	Did not complete	Did not complete

P14	Did not complete	Did not complete	Did not complete	Did not complete	Did not complete	Did not complete
P15	Did not complete	Did not complete	Did not complete	Did not complete	Did not complete	Did not complete
P16	Did not complete	Did not complete	Did not complete	Did not complete	Did not complete	Did not complete
P17	Did not complete	Did not complete	Did not complete	Did not complete	Did not complete	Did not complete
P18	Did not complete	Did not complete	Did not complete	Did not complete	Did not complete	Did not complete
P19	Yes	Yes	High School	None	Self-manage	Immediate whānau
P20	Yes – Breast Cancer	Yes	High School	None	Self-manage	Immediate whānau
P21	Yes – Type 1 Diabetes	Yes	High School	None	Self-manage	Immediate whānau
P22	No	Yes	High School	Some assistance	Healthcare professional	Immediate whānau
P23	Yes	Yes	High School	None	Self-manage	Immediate whānau
P24	Yes – Chronic pain	No	Trade/Certificate	Some assistance	Self-manage	Immediate whānau
P25	Yes – Sciatica	Yes	Did not complete	Some assistance	Self-manage	Immediate whānau
P26	No	No	High School	None	Self-manage	Immediate whānau
P27	Yes – Ventricular tachycardia	Yes	Did not complete	None	Self-manage	Immediate whānau
P28	No	No	University	None	Self-manage	Immediate whānau

P29	No	No	Trade/Certificate	None	Self-manage	Immediate whānau
P30	Did not complete	Did not complete	High School	Some assistance	Self-manage	Immediate whānau
